# Support with Impella versus intra-aortic balloon pump in acute myocardial infarction complicated by cardiogenic shock: A protocol for systematic review and meta-analysis

**DOI:** 10.1097/MD.0000000000025159

**Published:** 2021-03-26

**Authors:** Lingzhang Rao, Xianli Huang, Jinlan Luo

**Affiliations:** Department of Cardiology, Wuhan Wuchang Hospital, Wuchang Hospital Affiliated to Wuhan University of Science and Technology, Hubei, China.

**Keywords:** Acute myocardial infarction, cardiogenic shock, Impella, intra-aortic balloon counterpulsation, meta-analysis, protocol

## Abstract

**Background:**

The survival benefit and safety of Impella support versus intra-aortic balloon counterpulsation (IABP) in patients with acute myocardial infarction (AMI) complicated by cardiogenic shock were investigated in several observational trials that revealed mixed results. Thus, in order to provide new evidence-based medical evidence for clinical treatment, we undertook a meta-analysis to assess the efficacy and safety of Impella versus IABP in AMI complicated by cardiogenic shock.

**Methods:**

We will search the EMBASE, Web of Knowledge, PubMed, ClinicalTrials.gov, and Cochrane Library from inception to Mar 2021 to retrieve relevant studies. Two independent authors will extract the information from the selected studies. Disagreements will be resolved through a discussion with a third review author. The outcomes include mortality and complications. The quality of randomized trials will be assessed by Cochrane risk of bias tool for randomized controlled trials and the risk of bias in non-randomized studies - of Interventions for non-randomized, observational studies. Review Manager software (v 5.4; Cochrane Collaboration) will be used for the meta-analysis.

**Results:**

The present meta-analysis will compare the efficacy and safety of Impella versus IABP in AMI complicated by cardiogenic shock.

**Conclusions:**

The results of our review will be reported strictly following the PRISMA criteria and the review will add to the existing literature by showing compelling evidence and improved guidance in clinic settings.

**OSF registration number:**

10.17605/OSF.IO/SKEQ7.

**Ethics and dissemination:**

Ethical approval and patient consent are not required because this study is a literature-based study. This systematic review and meta-analysis will be published in a peer-reviewed journal.

## 1 Introduction

Cardiogenic shock is the most severe complication of acute myocardial infarction (AMI), with a mortality rate of around 50%. Urgent coronary revascularization and inotropes are the main form of treatment.^[1]^ Many patients with cardiogenic shock at the acute phase of myocardial infarction die rapidly, whereas some are easily weaned from their pharmacological support. A subset of patients show relative haemodynamic stability with initial treatment, but remain dependent on it; many of them ultimately die of multiorgan failure.^[2]^

Short-term mechanical circulatory support devices can be deployed to support the endangered circulation. Intra-aortic balloon counterpulsation (IABP) has been the most widely used mechanical circulatory support device for the last decades.^[3,4]^ Today, IABP usage has a class IIb recommendation in the American guidelines and a class III recommendation in the European guidelines. The lack of efficacy of the IABP is likely to be, at least partly, the reason for the observed increased usage of more potent mechanical circulatory devices.^[5]^

Furthermore, more powerful devices such as the Impella (Abiomed, Danvers, Massachusetts) were increasingly applied. The Impella is a micro-axial flow pump actively delivering blood from the left-ventricular into the ascending aorta and can augment cardiac output.^[6]^ Depending on the specific device, Impella provides a flow of up to 5 L/min, and it may increase mean arterial pressure and may reduce myocardial work. Experimental studies suggested left-ventricular unloading, an increase in cardiac output, and improved blood flow in the coronary arteries in Impella patients.^[7]^

The survival benefit and safety of Impella support versus IABP in patients with AMI complicated by cardiogenic shock were investigated in several observational trials that revealed mixed results.^[8–11]^ Previous studies have been limited in their ability to provide strong evidences, such as small sample size and inconsistent adherence to modern methodological research standards, making it difficult to draw meaningful conclusions from individual trials. Thus, in order to provide new evidence-based medical evidence for clinical treatment, we undertook a meta-analysis to assess the efficacy and safety of Impella versus IABP in AMI complicated by cardiogenic shock.

## 2 Materials and methods

### 2.1 Protocol registration

The prospective registration has been approved by the Open Science Framework (OSF) registries (https://osf.io/skeq7), and the registration number is 10.17605/OSF.IO/SKEQ7. The protocol was written following the Preferred Reporting Items for Systematic Reviews and Meta-Analyses Protocols statement guidelines.

### 2.2 Searching strategy

**Figure 1:**
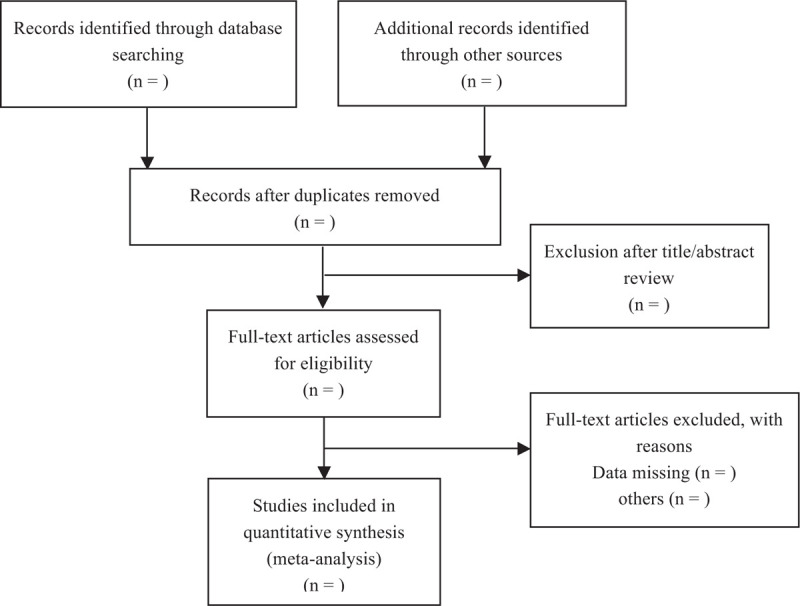
PRISMA flow diagram describing the selection process for relevant clinical trials used in this meta-analysis.

We will search the EMBASE, Web of Knowledge, PubMed, ClinicalTrials.gov, and Cochrane Library from inception to Mar 2021 to retrieve relevant studies using the following search terms: “Impella, intra-aortic balloon pump, IABP, mechanical circulatory support, acute myocardial infarction, AMI, cardiogenic shock.” No language restrictions will be applied. We will also search citations of relevant primary and review. Authors of abstract in the meeting will be further searched in PubMed for potential full articles. To minimize the risk of publication bias, we will conduct a comprehensive search that included strategies to find published and unpublished studies. The research summary of the screening flow chart is shown in Figure 1. Ethical approval is not necessary because the present meta-analysis will be performed based on previously published studies.

### 2.3 Eligibility criteria

Study included in this review have to meet all of the following inclusion criteria in the PICOS order:

(1) population: patients with AMI complicated by cardiogenic shock;
(2) intervention group (group 1): Impella group;
(3) comparison group (group 2): IABP group;
(4) outcome measures: at least one of the following outcome measures was reported: mortality and complications;
(5) study design: randomized controlled trial or observational study.

Biomechanical studies, in vitro studies, review articles, techniques, case reports, letters to the editor, and editorials are excluded.

### 2.4 Study selection

The first author will conduct a preliminary screening based on the title to eliminate any research not related to the topic. A log of excluded studies is kept with the rationale for exclusion. Subsequently, all remaining abstracts will be reviewed by the primary author, and the selection criteria are applied. Studies identified for full text review will be evaluated by 2 authors for inclusion in the study. Disagreements will be resolved through a discussion with a third review author. Journal titles and authors’ names will be not glossed over in the research selection process. A manual search of the bibliographies of included studies is performed to ensure that the overall search was comprehensive and complete.

### 2.5 Data extraction

The method of data extraction will follow the approach outlined by the Cochrane Handbook for Systematic Reviews of Interventions. Two independent authors will extract the following descriptive raw information from the selected studies: study characteristics such as the first author, publication year, study design, follow-up period; patient demographic details such as patients’ number, average age, and gender ratio. The outcomes include mortality and complications. Where disagreement in the collection of data occurs, this will be resolved through discussion. The corresponding author will be contacted and asked to provide the data if the SD is not reported. In the case of no response, the SD will be calculated from the available data according to the previously validated formula: (higher range value - lower range value)/4 or interquartile range/1.35. The highest SD will be used if the SD cannot be calculated using this approach. If necessary, we will abandon the extraction of incomplete data.

### 2.6 Statistical analysis

Review Manager software (v 5.4; Cochrane Collaboration) will be used for the meta-analysis. Continuous variables are extracted and analyzed to mean value ± SD. Standardized mean differences with a 95% confidence interval are assessed for continuous outcomes. The heterogeneity is assessed by using the *Q* test and *I*^2^ statistic. An *I*^2^ value of <25% is chosen to represent low heterogeneity and an *I*^2^ value of >75% to indicate high heterogeneity. All outcomes are pooled on random-effect model. A *P* value of < .05 is considered to be statistically significant.

### 2.7 Quality evaluation

The quality of randomized trials will be assessed by Cochrane risk of bias tool for randomized controlled trials and the risk of bias in non-randomized studies - of Interventions for non-randomized, observational studies. Each paper will be reviewed by one reviewer and verified by a second and disagreements will be resolved by discussion with a third reviewer. A meta-analysis will be conducted when 3 or more trials reported an outcome of interest. We also will perform the sensitivity analysis to evaluate whether the differences of study design had an impact on the overall estimate and data. Review Manager software (v 5.4; Cochrane Collaboration) will be conducted for statistical investigation and a funnel plot analysis will be drawn to assess the publication bias if there are more than 10 studies included.

## 3 Discussion

The survival benefit and safety of Impella support versus IABP in patients with AMI complicated by cardiogenic shock were investigated in several observational trials that revealed mixed results. Previous studies have been limited in their ability to provide strong evidences, such as small sample size and inconsistent adherence to modern methodological research standards, making it difficult to draw meaningful conclusions from individual trials. Thus, in order to provide new evidence-based medical evidence for clinical treatment, we undertook a meta-analysis to assess the efficacy and safety of Impella versus IABP in AMI complicated by cardiogenic shock. This is the first meta-anslysis of to investigate the efficacy and safety of Impella versus IABP in AMI complicated by cardiogenic shock. For this study, our review process will be very rigorous. And this article is a protocol of the systematic review and meta-analysis, which presents the detailed description of review implement. The results of our review will be reported strictly following the PRISMA criteria and the review will add to the existing literature by showing compelling evidence and improved guidance in clinic settings.

## Author contributions

**Conceptualization**: Lingzhang Rao, Xianli Huang.

**Data curation**: Lingzhang Rao, Xianli Huang.

**Formal analysis:** Lingzhang Rao, Xianli Huang.

**Funding acquisition:** Jinlan Luo.

**Investigation**: Lingzhang Rao, Xianli Huang.

**Methodology**: Lingzhang Rao, Xianli Huang.

**Project administration:** Jinlan Luo.

**Resources**: Jinlan Luo.

**Software**: Lingzhang Rao, Xianli Huang, Jinlan Luo.

**Supervision:** Jinlan Luo.

**Validation:** Xianli Huang.

**Visualization:** Lingzhang Rao.

**Writing – original draft**: Lingzhang Rao, Xianli Huang.

**Writing – review & editing:** Jinlan Luo.
